# Investigation of infraclavicular block success using the perfusion index: A randomized clinical trial

**DOI:** 10.17305/bjbms.2022.8214

**Published:** 2023-05-01

**Authors:** İbrahim Şeyhanlı, Mehmet Duran, Nezir Yılmaz, Hamza Nakır, Mevlüt Doğukan, Öznur Uludağ

**Affiliations:** 1 Anesthesia and Reanimation Department, Adıyaman Training and Research Hospital, Adıyaman, Turkey; 2 Anesthesiology and Reanimation Department, Faculty of Medicine, Adıyaman University, Adıyaman, Turkey

**Keywords:** Infraclavicular block, perfusion index (PI), peripheral nerve block, brachial plexus (BP)

## Abstract

The results of the pinprick and cold tests performed on the arm, forearm, and wrist-wrist areas of patients scheduled for upper extremity procedures are subjective and dependent on patient’s compliance. The purpose of this study was to determine whether the perfusion index (PI) could be used as an objective indicator to demonstrate block efficacy. Fifty patients between the ages of 18 and 65 years who were scheduled for upper extremity procedures and had an American Society of Anesthesiologists risk assessment class of I–II were included in this study. Infraclavicular block was performed on the patients using the peripheral nerve stimulation and ultrasonography. Preoperative and postoperative PI values were measured and recorded. The pinprick test took an average of 7.98 ± 1.49 minutes to turn positive, whereas the grade 3 of Modified Bromage Scale took an average of 11.08 ± 1.71 minutes. Differences between baseline values and perioperative values were found to be significantly different in the paired comparisons of the PI values. With 80% sensitivity and 80% specificity, increases in the PI value by or above 3.8 units were indicative for sensory block. With 84% sensitivity and 84% specificity, increases in the PI value by or above 3.9 units were indicative for grade 3 of Modified Bromage Scale in patients. It was determined that the PI is a faster, more reliable, and simpler technique than conventional methods for determining the efficacy of a block because of the vasodilatation that occurs before sensory and motor block.

## Introduction

A common anesthetic technique for procedures on the upper extremities is brachial plexus (BP) block. The infraclavicular technique has increased in popularity with the use of ultrasonography (USG) in the clinical settings, as it has a lower complication rate and is both practical and simple to use [1]. Its benefits over other forms of anesthesia such as peripheral nerve block include minimal to no effect on the respiratory and circulatory systems, relief of postoperative discomfort, and a shorter hospital stay [2].

Assessment of sensory and motor functions helps determine whether peripheral nerve block is successful. The traditional methods, such as pinprick test and Modified Bromage Scale (MBS), often utilized in this examination are subjective and dependent on the patient’s cooperation [3]. On the other hand, perfusion index (PI) represents the ratio of pulsatile to non-pulsatile blood flow as determined by a special pulse oximeter. It is believed that PI can be used to objectively assess changes in vascular tone and vasodilation induced by peripheral nerve block [4].

An increase in PI readings when peripheral nerve block is performed is due to vasodilation and an increase in blood flow in the extremity. Therefore, an increase in PI values can assess the indirect success of peripheral nerve block administered without requiring the patient’s cooperation [4].

In this study, the applicability of PI as an objective metric demonstrating the success and efficacy of infraclavicular block was evaluated.

## Materials and methods

The study was conducted at the Adıyaman University Research and Training Hospital between 01 August 2021 and 15 April 2022.

The study included 50 patients between the ages of 18 and 65 years who were in the American Society of Anesthesiologists (ASA) I–II group and were scheduled for upper extremity surgery (hand, wrist, and forearm). Patients with diabetes, peripheral vascular disease, allergies to the local anesthetics (LAs), alpha- or beta-blocker use, local infections at the site of the operation, suspicion of nerve injury found during a neurologic examination prior to surgery, and refusal to participate in the study were excluded from the study.

The time of block induction was defined as the infiltration of LA into the perineural area with a needle under ultrasound guidance. Peripheral oxygen saturation, heart rate, non-invasive arterial blood pressure values, and the PI values measured at both upper extremities were recorded in patients before block induction (0 min) and 5, 10, and 20 min after block completion.

Asepsis in the infraclavicular region was achieved when the patient was laid in the supine position with their head turned to the opposite side. The median, lateral, and posterior cords of the BP were visualized using a high-frequency linear ultrasound probe around the artery in the infraclavicular area. To perform the USG guided in-plane technique, a 22G 50-mm needle was used. A second confirmation was performed using a nerve stimulator (NS) by applying 0.2–0.8 mA electrical stimulation. After observing the motor response of each chord, perineural LA infiltration was administered to each cord. For this treatment, each patient received 20 mL of 0.5% bupivacaine (Buvicaine 0.5%, 5 mg/mL Polifarma) and 2% lidocaine (Jetmonal 2%, 20 mg/mL, Adeka, Turkey).

The sensory onset times of the block in all upper extremity areas, including the axillary nerve (lateral side of the upper arm), musculocutaneous nerve (lateral side of the forearm), radial nerve (dorsal part of hand at the 2nd metacarpophalangeal joint), median nerve (thenar eminence), ulnar nerve (little finger), and cutaneous nerves, were measured every 5 min until 30 min after the last injection (medial side of the upper arm and the medial side of the forearm). Three minutes after the block was induced, the pinprick test was used to assess and record the degree of sensory blockage in the affected arm (0: no sensory block; 1: sensation of touch present, no pain; 2: no sensation of touch, no pain). The PI readings were recorded at this exact time, along with the minute the pinprick test was positive.

Every 5 min, until the 30 min, 5 motor nerves were evaluated for the motor block: the musculocutaneous (elbow flexion), radial (thumb abduction), median (third digit flexion), ulnar (fifth digit flexion), and the axillary nerve (arm abduction). The results were then compared with those of the other arm. Three minutes after the block was induced, the degree of motor blockade in the affected arm was measured and recorded every minute using the MBS (0: no block, the patient can lift the arm; 1: motor strength reduced, but the arm can move; 2: the arm is immobile, but the digits can move; 3: complete block, no movement in the arm or hand). The minute the grade reached 3 on the MBS was noted, as well as the PI values at that exact moment.

The application of peripheral nerve blocks, the inspection of sensory-motor blocks, and the assessment of PI values were performed by several researchers. Both the patients and researchers were blinded to the examination findings and measured values.

### Ethical statement

This study was conducted in accordance with the principles of the Helsinki Declaration and all applicable national regulations and institutional policies (as revised in 2013). This study was reviewed and approved by Adıyaman University Clinical Studies Ethics Committee (decision date: 23/06/2020, decision number: 2020/6-18) and registered on clinicaltrials.gov (NCT05234541). Written informed consent was obtained from all participants included in this study.

### Statistical analysis

The sample size was calculated using MedCalc Software version 14 (MedCalc Software bvba, Ostend, Belgium) to find the null hypothesis with the area under the receiver operating characteristic (AUROC) curve of 0.5 and AUROC of 0.80. We calculated a minimum of 45 patients for a study power of 80% and an error of 0.05 because it is assumed that the success rate of the infraclavicular block is more than 80%.

The data collected in the study were analyzed using the SPSS 22.0 (Statistical Package for the Social Sciences) program. The normality of the distributions of the variables was tested using visual (histograms and probability plots) and analytical (Shapiro–Wilk test) methods. Levene’s test was used to check the homogeneity of the group variances. The mean differences between two groups were analyzed with Student’s t test. In the intragroup comparisons of time-related changes, the Friedman test was used for the non-normally distributed variables. For the post hoc comparisons, Wilcoxon signed-rank test was applied separately for pair of groups. Each output was reported by applying Bonferroni correction. The receiver operating characteristic (ROC) curve was used to examine the recorded values for our outcome criteria in terms of their diagnostic value. The Hanley and McNeil method was used to calculate the ROC curve and the area under the ROC curve (AUC). AUC values close to 1.0 were interpreted as indicating an improvement in prediction accuracy. The type 1 error rate of 5% was accepted for significance in all statistical analyses.

## Results

**Figure 1.**
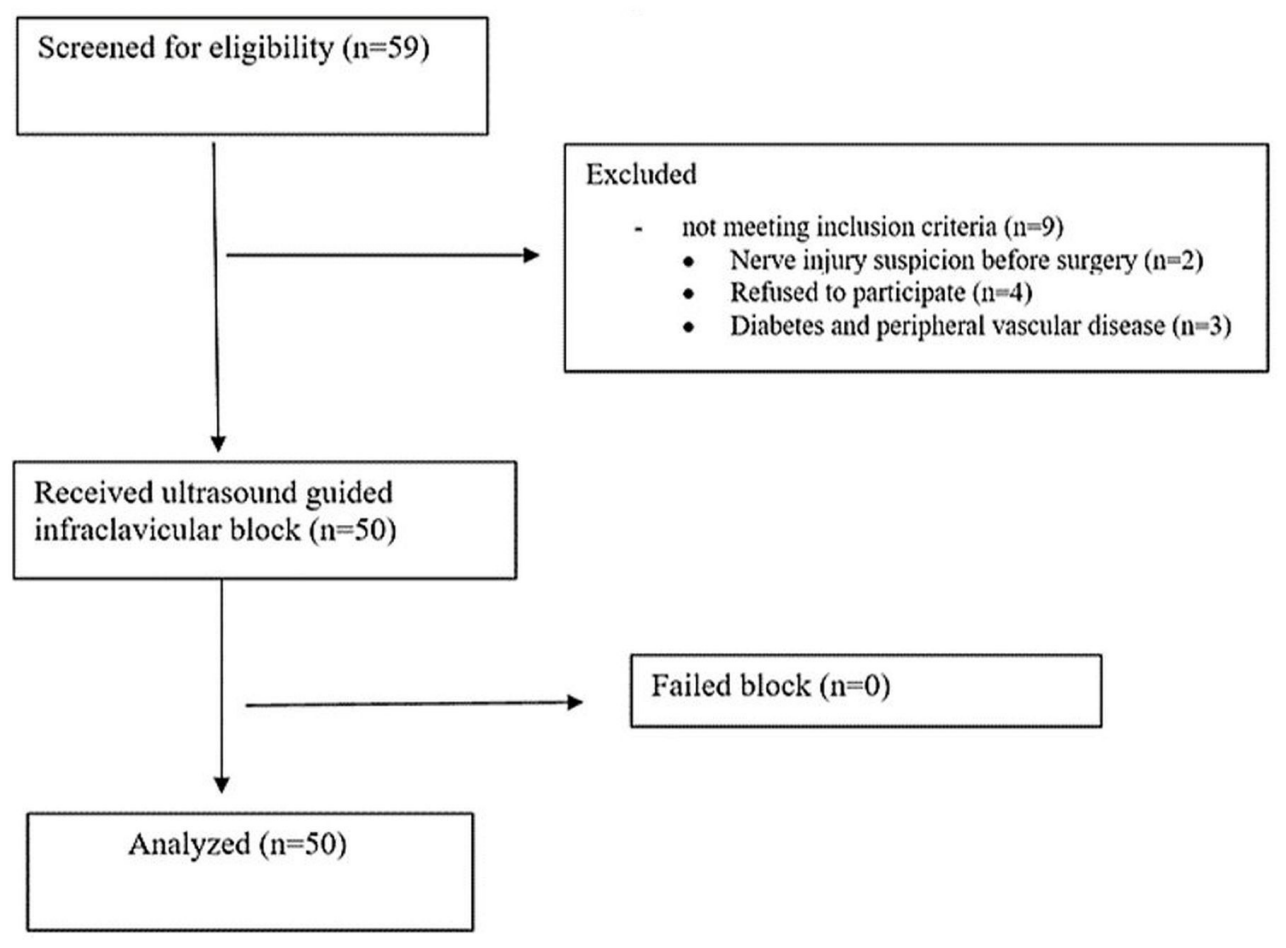
Patients’ enrolment flowchart.

Fifty-nine patients scheduled for upper limb orthopedic surgery under ultrasound-guided infraclavicular block were screened for eligibility; nine patients were excluded for not meeting our inclusion criteria. Fifty participants received infraclavicular nerve block, none of whom had a completely failed block. All included patients were available for the final analysis (Figure 1).

The study included 50 patients scheduled for upper extremity surgery. Of them, 19 patients had a distal radius fracture, 17 a radial ulna fracture, 5 a metacarpal fracture, 9 a tendon injury, 4 a carpal tunnel syndrome, and 3 a soft tissue tumor. All blocks induced using the infraclavicular approach using USG and NS resulted in success. The PI, derived from the photoplethysmography signal and representing the ratio of pulsatile to non-pulsatile light absorption or reflection of the photoplethysmography signal, was measured by a pulse oximeter sensor attached to a finger of the blocked upper extremity. Among the 50 patients who were included, 14 were female and 36 were male. The demographic characteristics of the included patients are shown in Table 1.

**Table 1:** Demographic characteristics of the patients

**Demographic characteristics**	**n (%) or X±SD**
Number of patients	50
Sex (female/male)	14 (28%) / 36 (72%)
Age (years)	34.1 ± 13.5
BMI (kg/m^2^)	26 ± 5
ASA (I/II)	31 (62%) / 19 (38%)
Duration of operation (h)	1.54 ± 0.54

BMI: Body mass index; ASA: American Society of Anesthesiologists; SD: Standard deviation.

The mean and standard deviation values of the hemodynamic parameters of the patients at 0, 10, 20, 30 and 60 min following the procedure are shown in Table 2. The systolic arterial pressure values of the patients at the 0 min were significantly higher than their systolic arterial pressure values measured at other times (*p* < 0.01). The diastolic arterial pressure values of the patients at 0 min were significantly higher than their diastolic arterial pressure values measured at other times (*p* < 0.01). There was no significant difference in their heart rate and peripheral oxygen saturation values.

**Table 2:** Time-based hemodynamic parameter—mean values

**Minute**	**0**	**10**	**20**	**30**	**60**	***p***
SAP	118 ± 9.3	114 ± 7.9	114 ± 7.1	113 ± 6.8	113 ± 5.6	**<0.001**
DAP	74 ± 7.2	70 ± 6.3	69 ± 4.3	70 ± 5.9	70 ± 5.4	**<0.001**
HR	75 ± 8.2	75 ± 7.4	75 ± 6.8	74 ± 6.9	74 ± 7.1	0.054
SPO_2_	98 ± 1.4	98 ± 1.3	98 ± 1.3	98 ± 1.3	98 ± 1.2	0.123

Bold values indicate statistical significance. SAP: Systolic arterial pressure; DAP: Diastolic arterial pressure; HR: Heart rate; SPO_2_: Peripheral oxygen saturation.

**Figure 2.**
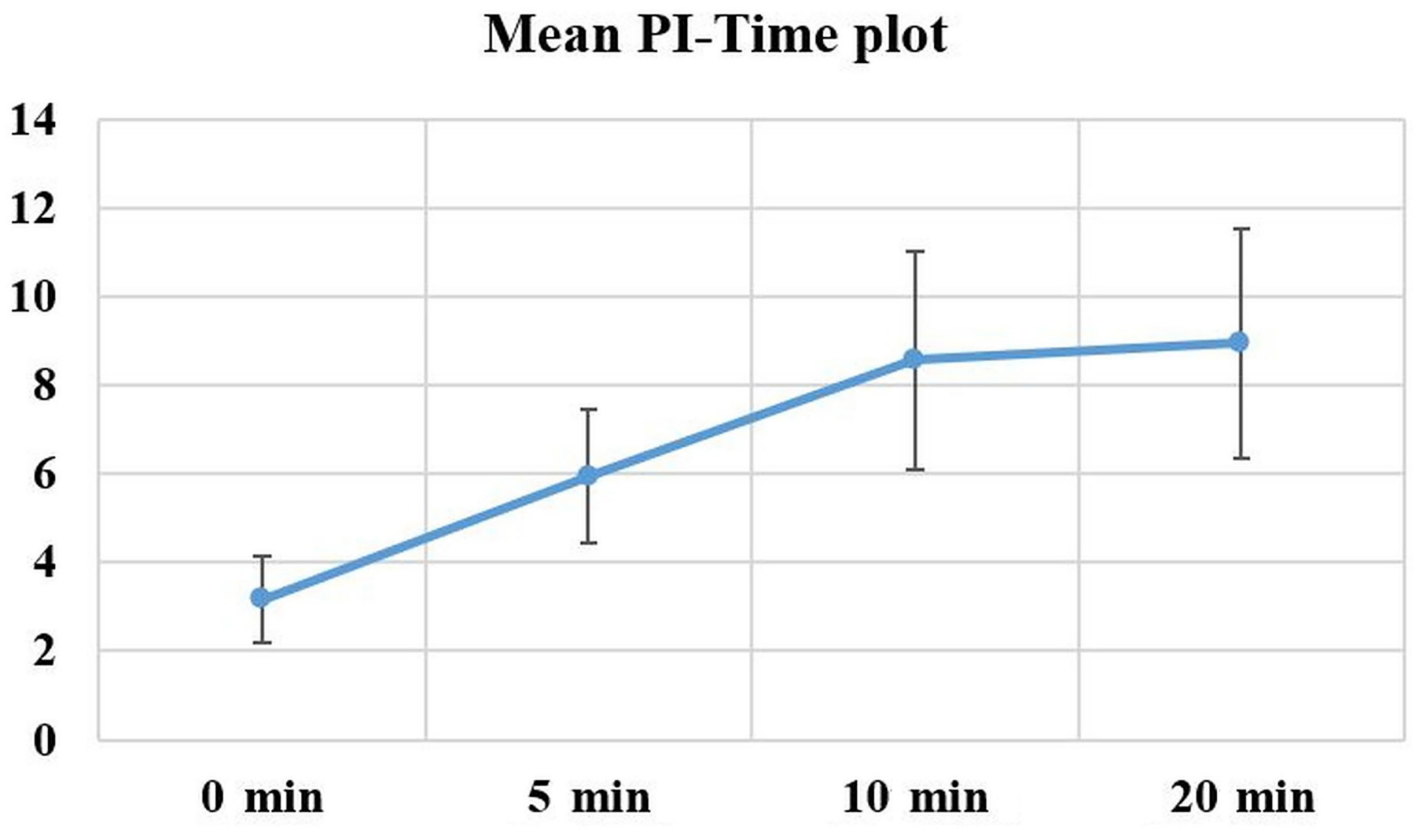
**The graphic of mean PI value changes by time.** PI: Perfusion index.

The mean PI values of the patients measure before block induction (0 min) and 5, 10, and 20 min after block induction are shown in Figure 2. The 0 min and 5 min mean PI values of the patients were significantly different in comparison to their values measured at all other times (*p* < 0.01). The mean PI values of the patients increased based on time by 105% from the baseline value (0 min) to 5 min, 42.7% from 5 min to 10 min, and 1.8% from 10 min to 20 min.

**Figure 3.**
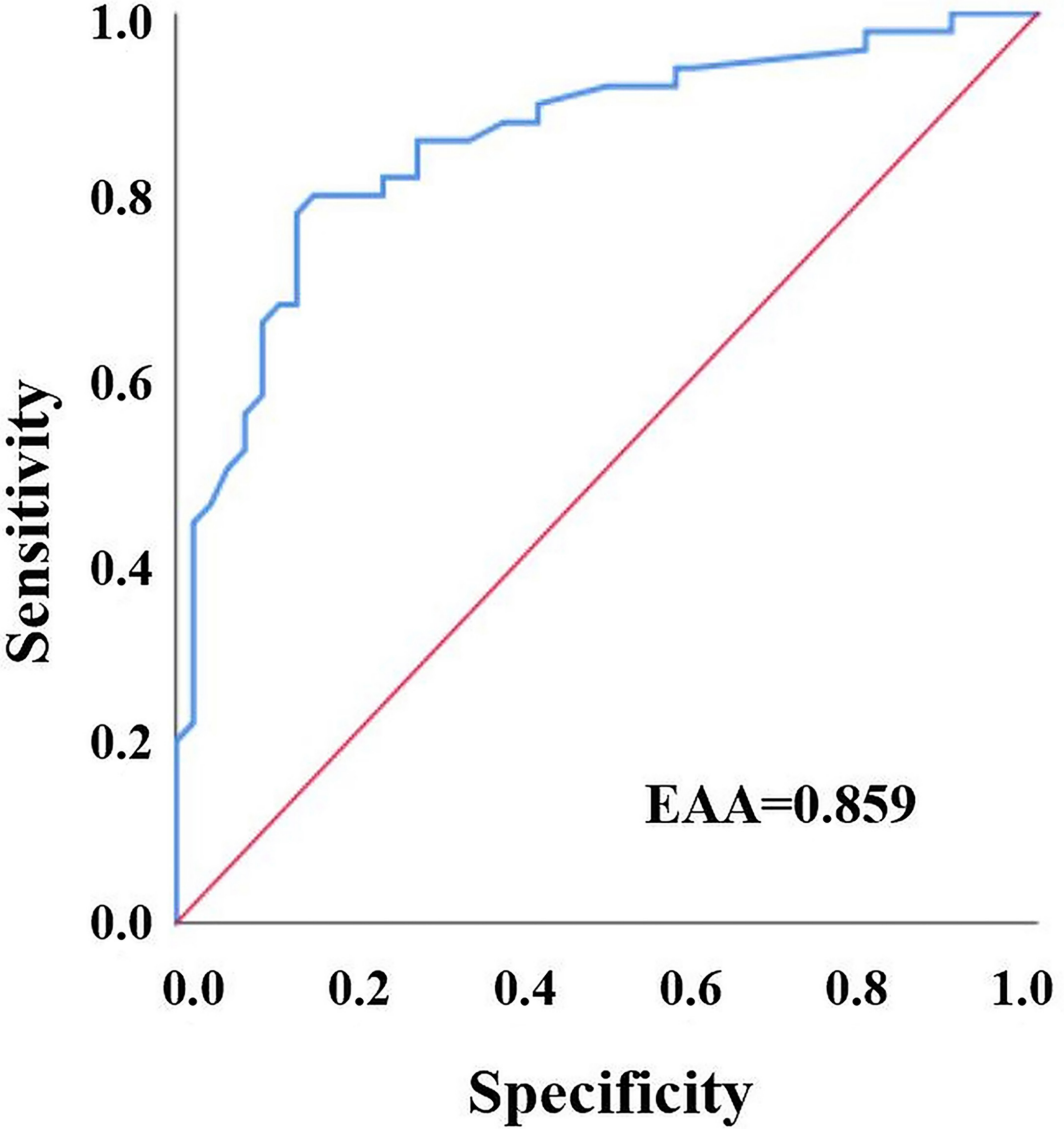
**Receiver operating characteristics curve for **Δ**PI-sensory block relationship.** PI: Perfusion index; AUC: Area under the curve.

The pinprick test results of the patients became positive at a mean time of 7.98 ± 1.49 min. The mean PI value at the onset of pinprick test positivity was 8.5 ± 2.46. To confirm the diagnostic accuracy of the sensory block (pinprick test), the differences of each PI value from the baseline PI values (ΔPI) were recorded starting at 5 min. A ROC curve was drawn for each value, and their predictive values were separately examined (Figure 3). In the prediction of sensory block, for ΔPI, the AUROC was 0.859, whereas the optimal cutoff value was 3.8. Accordingly, an increase in PI values by 3.8 units or more could predict sensory block development with 80% sensitivity and 80% specificity (*p* < 0.001) (Table 3).

**Table 3:** Receiver operating characteristics curve statistics for ΔPI-sensory and motor block relationships

	**AUC (95% CI)**	**Cutoff value**	**Sensitivity**	**Specificity**	***p***
**Sensory block** **(Pinprick test)**	0.859 (0.784–0.933)	3.8	82%	82%	**<0.001**
**Motor block** **(MBS grade 3)**	0.895 (0.832–0.957)	3.9	84%	84%	**<0.001**

Bold values indicate statistical significance. AUC: Area under the curve; CI: Confidence interval; MBS: Modified Bromage Scale; PI: Perfusion index.

**Figure 4.**
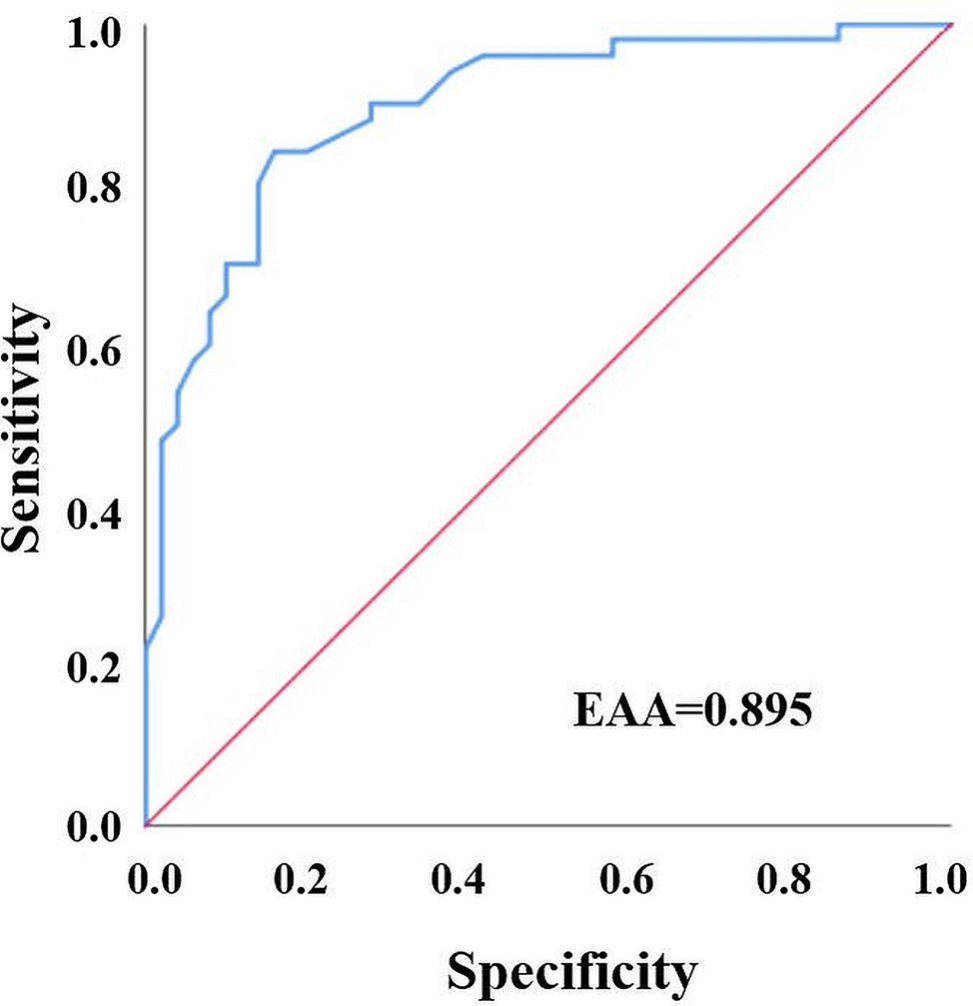
**Receiver operating characteristics curve for **Δ**PI-motor block (Modified Bromage Scale grade 3) relationship.** PI: Perfusion index; AUC: Area under the curve.

Grade 3 of the MBS became positive at a mean time of 11.08 ± 1.71 min. The mean value of PI where this test became positive was 8.86 ± 2.6. To confirm the diagnostic accuracy of the motor block (MBS grade 3), the differences of each PI value from the base PI values (ΔPI) were recorded starting at 3 min. An ROC curve was drawn for each value, and their predictive values were separately examined (Figure 4). In the prediction of grade 3 of the MBS, for ΔPI, the AUROC was 0.895, whereas the optimal cutoff value was 3.9. Accordingly, an increase in PI values by 3.9 units or more could predict motor block (MBS grade 3) development with 84% sensitivity and 84% specificity (*p* < 0.001) (Table 3).

## Discussion

Over the years, several techniques have been used to achieve successful and safe peripheral block application. The paresthesia technique is a blind technique based on direct contact with nerve fibers. This technique is based on the perception of stimulation sensation and is a subjective technique that can be confusing in most cases. The NS technique, on the other hand, is based on finding the inverse relationship between current intensity and distance to show the proximity of the needle to the nerve. The NS technique has been shown to be a highly effective technique for determining the correct needle position for induction of local anesthesia [5]. USG allows direct and real-time visualization of nerve structures, needle position, and LAs and is therefore prevalently used in peripheral nerve blocks. In studies where block implementations have been carried out under USG guidance, the use of USG was associated with lower use of LA, higher block success rates, lower complication rates, and lower opioid consumption [6, 7]. In this study, to reduce complication rates and increase the success of the block, USG and NS were used together for guidance. Using this method, infraclavicular blocks were successfully performed in all 50 patients in this study. No local or systemic complications were encountered.

In our study, after evaluating the vital signs of the patients by monitoring their values, sedoanalgesia was induced with 1 mg midazolam and 50 µg fentanyl before the block procedure. Studies have shown that BP blocks can be performed in awake or mildly sedated patients by an experienced anesthesiologist [8]. Sedoanalgesia that is induced before regional anesthesia (RA) reduces fears of surgery in patients and the anxiety that could occur in relation to these fears, and it positively affects patient comfort and adjustment in the perioperative, intraoperative, and postoperative period [9]. Anxiolytics and narcotic analgesic agents are frequently used for sedation. It has been reported that the use of these agents as bolus and maintenance infusion accelerates the onset of block in peripheral nerve blocks and prolongs the duration of the block [10]. However, no effect of a single dose mild sedation was found on the onset and duration of block. It is suggested that the reduction in the systolic and diastolic arterial pressure values of the patients in our study was associated with the sedoanalgesia that was induced.

Successful BP block is associated with reduced vascular tone and increased blood flow. PI represents the ratio of the pulsatile and non-pulsatile components of peripheral blood flow to each other. During vasodilation, the increase in the pulsatile flow leads to an increase in the PI value. Available data demonstrate that PI is a sensitive parameter to express the vasomotor tone loss induced by LAs. Therefore, PI can be considered an objective measure of peripheral perfusion, which can predict the success of peripheral block [11]. In our study, the PI values measured in the extremity in which the block was induced at 5 min were significantly higher than those measured before the block induction. Similarly, 10 min PI values were significantly higher than 5 min values, and 20 min values were significantly higher than 10 values. It is thought that these significant differences in the PI values can be explained by the sympathetic blockade that forms after block induction, and by the vasomotor tone loss that develops with this blockade [12]. Candan et al. [13] reported that PI values at 5 min measured in the extremity where an infraclavicular block was induced were 132% higher than baseline PI values. In another study, Galvin et al. [14] determined a 155% increase in PI at 10 min of axillary blockade and at 12 min of sciatic blockade by using 1.5% mepivacaine. Kuş et al. [15] reported a 120% increase in PI at 10 min of infraclavicular blockade, while Abdelnasser et al. [16] reported a mean increase of 151% in PI values at 10 min after inducing supraclavicular blockade. In our study, in comparison to the baseline PI values, we found an increase of 105% at 5 min and an increase of 171% at 10 min. It is suggested that the different PI value change ratios by time reported in the cited studies were associated with the volume and type of LAs that were used in these studies.

In cases of vasodilatation, a relative increase in the pulsatile blood flow leads to an increase in the PI value. Thus, PI is considered an objective measure for predicting the peripheral block success. The efficacy of PI in predicting the peripheral block success has been demonstrated in BP blocks [11, 15], axillary BP blocks [13], interscalene BP blocks [12], sciatic nerve blocks [14], and supraclavicular BP blocks [16]. Of these studies, Kuş et al. [15] showed that PI, a measure of peripheral perfusion, was a reliable and objective method for assessing the efficacy of infraclavicular blockade, but they did not report any cutoff value for PI. In the study by Galvin et al. [14], a 1.55-fold increase in PI was considered a successful block. Abdelnasser et al. [16] stated that PI can be used to predict the supraclavicular BP block success, and that an increase of 3.3 units or more in the PI value had 100% sensitivity and specificity for motor block development. However, they did not provide any cutoff value for sensory block. Bereket et al. [17] reported that the infraclavicular BP block success can be determined based on PI, and an increase of 9.2 units or more in PI had 54.5% specificity and 96.6% sensitivity for block development. However, they did not provide separate cutoff values for the onset of sensory block and motor block.

Our study aimed to present the diagnostic relationships between sensory block and motor block development and PI values and to determine the optimal cutoff points for PI values. Using the pinprick test, sensory block was detected at 7.98 ± 1.49 min. The mean PI value at which the test became positive was 8.5 ± 2.46. In the ROC analyses, it was seen that an increase of 3.8 units or more in the PI value calculated starting at 5 min after block induction could predict the development of sensory block with 82% sensitivity and 82% specificity. Using MBS grade 3 tests, motor block was detected at 11.08 ± 1.71 min. The mean PI value at which the test became positive was 8.86 ± 2.6. It was found that an increase of 3.9 units or more in the PI value calculated starting at 3 min after block induction could predict the development of motor block on the level of MBS grade 3 with 84% sensitivity and 84% specificity.

The literature review revealed that, while there were numerous studies in which PI values demonstrated BP block success, few studies had separately analyzed sensory and motor blocks using PI values. In this sense, our study is the first example of this in the literature. As stated above, the PI cutoff values reported in different studies in the literature differ from each other. It is believed that these differences were caused by differences in the methodologies of the studies, such as the type and volume of LA used, the type of blockades applied, and the experience of the practitioners.

Some limitations of our study should be mentioned. Patient-related differences in the assessment of block success may have resulted from the subjective methods we used to evaluate the establishment of the sensory and motor blocks. The fact that the study was single-centered and all infraclavicular blocks were performed by a single anesthesiologist can also be considered as limitation. Finally, PI values vary with age. It is predicted that different age groups have different cutoff values [18]. Our results are limited to patients aged 18–65 years.

## Conclusion

PI is a useful and objective metric in the prediction of the infraclavicular PB block success. It was found that block success may be predicted by degrees of increase of 3.8 or higher for sensory block and 3.9 or higher for motor block.
